# Fast free energy estimates from λ-dynamics with bias-updated Gibbs sampling

**DOI:** 10.1038/s41467-023-44208-9

**Published:** 2023-12-21

**Authors:** Michael T. Robo, Ryan L. Hayes, Xinqiang Ding, Brian Pulawski, Jonah Z. Vilseck

**Affiliations:** 1 Department of Biochemistry and Molecular Biology, Indiana University School of Medicine, Indianapolis, IN 46202 USA; 2 Center for Computational Biology and Bioinformatics, Indiana University School of Medicine, Indianapolis, IN 46202 USA; 3 Chemical and Biomolecular Engineering, University of California, Irvine, California, 92617 USA; 4 Pharmaceutical Sciences, University of California, Irvine, CA 92617 USA; 5 Department of Chemistry, Massachusetts Institute of Technology, Cambridge, MA 02139 USA; 6 Present Address: Indiana Biosciences Research Institute, 1210 Waterway Blvd Ste. 2000, Indianapolis, IN 46202 USA; 7 Present Address: Department of Chemistry, Tufts University, Medford, MA 02144 USA

**Keywords:** Theoretical chemistry, Method development

## Abstract

Relative binding free energy calculations have become an integral computational tool for lead optimization in structure-based drug design. Classical alchemical methods, including free energy perturbation or thermodynamic integration, compute relative free energy differences by transforming one molecule into another. However, these methods have high operational costs due to the need to perform many pairwise perturbations independently. To reduce costs and accelerate molecular design workflows, we present a method called λ-dynamics with bias-updated Gibbs sampling. This method uses dynamic biases to continuously sample between multiple ligand analogues collectively within a single simulation. We show that many relative binding free energies can be determined quickly with this approach without compromising accuracy. For five benchmark systems, agreement to experiment is high, with root mean square errors near or below 1.0 kcal mol^−1^. Free energy results are consistent with other computational approaches and within statistical noise of both methods (0.4 kcal mol^−1^ or less). Notably, large efficiency gains over thermodynamic integration of 18–66-fold for small perturbations and 100–200-fold for whole aromatic ring substitutions are observed. The rapid determination of relative binding free energies will enable larger chemical spaces to be more readily explored and structure-based drug design to be accelerated.

## Abstract (web summary)

Calculations of relative binding free energy are crucial for lead optimization in structure-based drug design, but classical methods are computationally expensive. Here, the authors describe a more efficient method for calculating the free energy that is as accurate as thermodynamic integration.

## Introduction

**Fig. 1:**
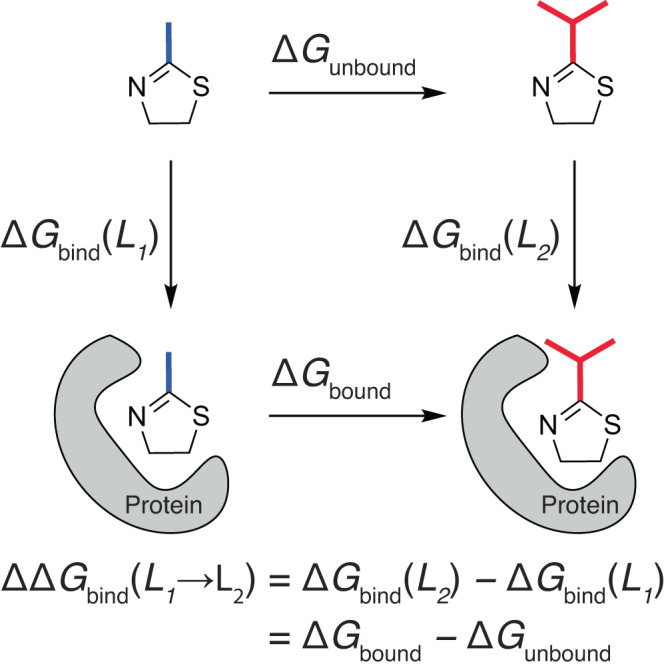
A thermodynamic cycle for computing relative binding free energies. Two ligands (*L*_1_ and *L*_2_) with different substituents (represented as a methyl group in blue or an iso-propyl group in red) are represented as bound to a protein (in gray) or unbound in solution. Relative binding free energies between *L*_1_ and *L*_2_ can be computed by alchemically transforming *L*_1_ into *L*_2_ in bound and unbound states.

Relative binding free energy (RBFE) calculations have emerged as a promising tool for the lead optimization of small molecule pharmaceuticals^1–3^. In an RBFE calculation, a small molecule bound to a protein target is alchemically transformed into a different small molecule, such as an analog formed by modifying one or more functional groups of the lead compound. The relative difference in free energies of binding (ΔΔ*G*_bind_) between the two molecules can then be calculated using a thermodynamic cycle (Fig. 1)^4^. Compared to methods such as molecular docking, RBFE calculations have shown significantly improved correlation between computed and experimental binding affinities, with errors of roughly 1 kcal mol^−1^ or less for state-of-the-art calculations^5–8^. Although too high to eliminate the need for experiment entirely, this degree of accuracy is low enough to separate compounds with stronger versus weaker binding affinities and efficiently prioritize molecules for experimental investigation^9,10^. Using stochastic simulations, Mobley and Klimovich quantified the effect that this computational prioritization can have on a drug discovery project. They estimate that RBFE calculations with an average of 1.0 kcal mol^−1^ of error to experiment can improve the odds of identifying a tenfold potency boost by a factor of 5^11^. When optimizing lead compounds for other drug-like properties, RBFE calculations can also be used to filter out compound modifications that might negatively affect potency^7^.

While many methods of RBFE calculations exist, the most commonly used methods are free energy perturbation (FEP) or thermodynamic integration (TI) coupled with the multistate Bennett acceptance ratio (MBAR) free energy estimator^12–15^. With these methods, an alchemical coupling parameter, called *λ*, is used to alchemically transform one molecule into another. To ensure sufficient phase space overlap exists between *λ* states and to achieve convergence in computed free energy differences, many intermediate discrete *λ* states are also defined (typically 10–20) that span a range of *λ* values between 0 and 1, the two molecule end states of interest^4,7,9^. Molecular dynamics (MD) simulations are performed at each of these discrete *λ* states, *λ* values held constant for the duration of the simulation, and the MD trajectories are then postprocessed to calculate the final free energy difference. Though effective, FEP and TI calculations require considerable computational resources, are exclusively pairwise, and are inherently unable to evaluate more than one RBFE at a time. For example, in a typical FEP/TI experiment^5,7,8,16^, 11 discrete *λ* states may be used to model a single perturbation, requiring 11 MD trajectories of 5–20 ns per *λ* window to be run, which amounts to a total of 55–220 ns of simulation time for a single RBFE result^6,16^. Longer simulations or additional windows may be needed for more challenging perturbations, such as ring additions or polar-to-non-polar transformations^17^. Further compounding computational costs, recommended best practices for investigating large sets of multiple ligands with FEP/TI necessitates the use of redundant calculations to provide improved accuracy around closed perturbation cycles^18–20^. Although the recent adoption of running MD simulations on graphical processing units (GPUs) has accelerated computational throughput and facilitated routine employment of RBFE calculations on large, parallel high-performance computing (HPC) resources for drug discovery^21–23^, costs per RBFE calculation remain high.

Driven by the high cost of pairwise RBFE calculations, many groups have investigated alternative methods to perform RBFE calculations with the aim of achieving comparable accuracy with lower computational costs per computed RBFE. Non-equilibrium switching free energy calculations have seen renewed interest^24–27^. Mostly run in a pairwise manner, these calculations require only ca. 20–40 ns per transformation and are highly parallelizable^24,27^, making them good candidates for HPC or cloud computing. A variety of expanded ensemble methods have also grown in popularity^28–32^. *λ*-dynamics (*λ*D)^33,34^, enveloping distribution sampling^35–37^, and *λ*-local elevation umbrella sampling methods^38–40^, to name a few, have all sought to calculate free energy differences between multiple thermodynamic states within a single calculation to increase efficiency through improved scalability.

**Fig. 2:**
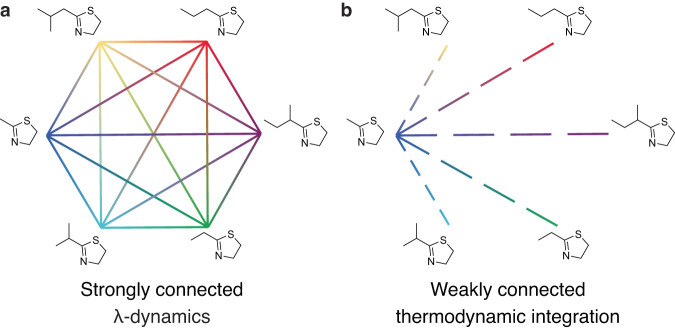
Alchemical perturbation connection graphs. **a** *λ*D-based methods sample a strongly connected graph of ligand end states. Efficiency gains obtained with *λ*D-based methods over traditional TI or FEP free energy methods originate from two key sources: (1) all physical and intermediate *λ* states are sampled within a single simulation (represented as solid lines) and (2) multiple ligands can be sampled simultaneously (represented as different colors). **b** TI or FEP methods sample a weakly connected graph via pairwise perturbations (commonly referred to as a “star map” when run without redundant calculations for cycle closure) and require many intermediate simulations to be run (represented as dashed lines).

In a conventional *λ*D simulation, *λ* is treated as a continuous parameter and its value can change dynamically in conjunction with the coordinates of an MD simulation, using extended Lagrangian methods^34,41^. Sampling of multiple ligand end states or sampling of many substituents at two or more sites of substitution are both feasible with multisite *λ*-dynamics (MSλD) via holonomic constraints^34,42^. Hence, multiple RBFEs can be computed from a single *λ*D simulation, lending large efficiency gains over conventional approaches. Recent benchmarks have shown that single-site perturbations can be performed with cost savings in the range of 3–5.4 times better than TI/MBAR^6^. Advantageously, sampling multiple ligand end states with *λ*D also allows alchemical transitions to occur from one end state to any other end state—forming a connection network termed “strongly connected” in graph theory—without the need for redundant calculations to form cycle closure connections, as commonly performed for FEP/TI (Fig. 2)^18,20^. Over the past few years, a variety of developments have been introduced to expand the utility of *λ*D for drug discovery, including an Adaptive Landscape Flattening (ALF) algorithm for automated bias determination^41,43^, a Potts model-based estimator for computing free energy differences and intersite couplings^44^, an accelerated GPU engine^45^, and an alternative *λ* sampling strategy using Gibbs sampling, a Markov chain Monte Carlo algorithm^46,47^. As discussed in-depth in “Methods”, this work builds upon this latter development of the discrete Gibbs sampler *λ*-dynamics (d-GSλD) method^47^.

To sample multiple ligands collectively within a single *λ*D simulation, however, free energy barriers between ligand end states in *λ*-space must first be flattened. This can be accomplished by identifying and incorporating a variety of biasing potentials into a *λ*D simulation^41^. These biases flatten intermediate free energy barriers and ensure ligand end states have equivalent free energies to facilitate rapid transitioning between end states. Though effective, a non-negligible amount of simulation time must be devoted to determining these biases prior to production sampling, e.g., recent *λ*D simulations have used 20–50 ns for bias determination^6,44,48,49^. With the advent of d-GSλD, discrete *λ* states can also be used to propagate alchemical transformations while maintaining the ability to sample all *λ* states within a single simulation. This approach works by forming two conditional distributions, *P*(*X* | *λ*) and *P*(*λ* | *X*), from the desired joint distribution of atomic coordinates, *X*, and alchemical states, *λ*, (*P*(*X*, *λ*)). Sequential sampling of these conditional distributions is then performed with Gibbs sampling to generate new values of *X* and *λ* at time *t*^32,47^. Molecular dynamics is used to sample *X* at a fixed *λ* state, while *λ* is sampled by calculating the potential energy of every *λ* state with a fixed set of atomic coordinates and selecting the next *λ* state proportional to its probability from a probability distribution using a pseudorandom number generator^47^. In d-GSλD, the use of discrete *λ* states is advantageous because conventional *λ*D biasing potentials can be simplified from a functional form into a single scalar value per discrete *λ* state. Biases added to individual *λ* states flatten energy barriers and facilitate stochastic transitions to different *λ* states over the course of a d-GSλD simulation. Although this does not remove the need to identify appropriate biases prior to production sampling, this approach reduces the amount of time needed to identify biases to 5–10 ns, on average. Highly accurate d-GSλD free energy estimates can then be obtained with MBAR^15,47,50^. Nonetheless, the computational cost of identifying biases for d-GSλD or *λ*D reduces the efficiency, throughput, and cost advantages of both methods. Thus, this work was motivated to try to eliminate these costs and accelerate RBFE calculations by removing the need to identify biases prior to production sampling. If such “biasing runs” could be avoided, we estimate that *λ*D-based methods could screen hundreds of compound analogs at a fraction of the cost of FEP/TI methods for drug discovery.

In this report, we implement the use of continuous bias updates in conjunction with discrete Gibbs sampler *λ*-dynamics to achieve rapid and accurate RBFE estimates. We refer to this method as *λ*-dynamics with bias-updated Gibbs sampling (LaDyBUGS). In contrast to the static biases used with d-GSλD, which were determined with a Wang–Landau-like algorithm^47^, LaDyBUGS uses an aggressive dynamic bias that changes and continuously drives the system to sample different *λ* states. This avoids the need to run separate simulations for bias determination prior to production sampling and continually refocuses sampling towards the least visited *λ* states to provide exceptionally smooth sampling of all *λ* states. FastMBAR, a GPU implementation of MBAR, is used for rapid free energy determination and on-the-fly bias refinement^50^. For five protein–ligand benchmark systems, we observe large efficiency gains of 18–66-fold improvements with LaDyBUGS compared to TI/MBAR without compromising accuracy in the predicted ΔΔ*G*_bind_ results. Larger efficiency gains (100–200-fold) are also observed for two systems involving more challenging perturbations of whole aromatic rings, where enhanced sampling in LaDyBUGS overcomes observed sampling limitations in TI/MBAR. In the following “Results and Discussion”, we evaluate LaDyBUGS’ performance in terms of accuracy compared to the experiment and efficiency compared to TI/MBAR, as implemented in OpenMM^51^. In “Methods”, we describe the workflow of LaDyBUGS as well as our computational procedure.

## Results and discussion

**Fig. 3:**
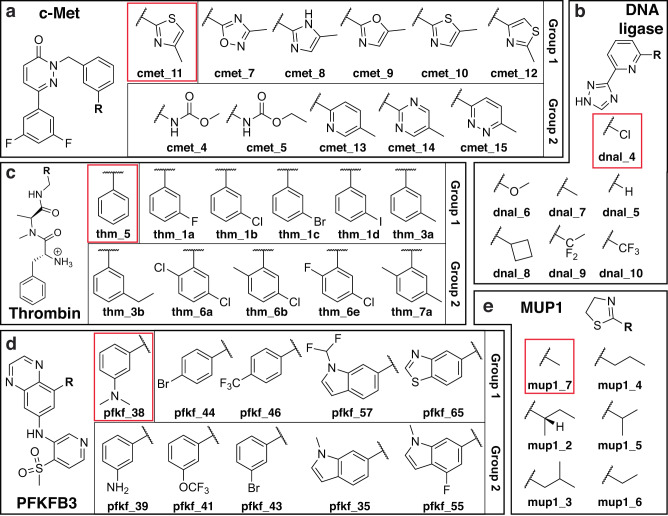
Protein–ligand benchmark systems. Five systems with 45 total ligands were used to evaluate and compare LaDyBUGS and TI/MBAR. LaDyBUGS calculations evaluated several ligands within a single simulation, and compounds were grouped in the following panels: **a** c-Met ligands, **b** DNA ligase ligands, **c** thrombin ligands, **d** PFKFB3 ligands, and **e** MUP1 ligands. For the TI/MBAR calculations, a star map of pairwise perturbations was utilized (Fig. 2). All relative free energy differences were calculated with respect to a reference compound for each system (highlighted in red). Ligand numbering was kept consistent with original experimental reports (c-Met^54^, DNA ligase^53^, thrombin^55^, and PFKFB3^56^) or a previous benchmark study (MUP1^52^).

Our goal in evaluating LaDyBUGS was to demonstrate that it provides comparable accuracy to classical methods for RBFE calculation with significant improvements in efficiency and cost savings. To that end, we selected five literature examples to benchmark LaDyBUGS performance: major urinary protein 1 (MUP1)^52^, DNA ligase^53^, c-Met kinase (c-Met)^54^, thrombin^55^, and 6-phosphofructo-2-kinase/fructose-2,6-biphosphatase 3 (PFKFB3)^56^. These systems have been featured in previous benchmarking studies of FEP+ and non-equilibrium switching^5,16,24,52^. In total, binding free energies were calculated for 45 different ligands: 6 for MUP1, 7 for DNA ligase, 11 for c-Met, 11 for thrombin, and 10 for PFKFB3 (Fig. 3). To avoid the complications of charge-changing perturbations^5,57,58^, only ligands with neutral alchemical substituents were included in this study. We also performed symmetric methyl perturbations to ensure no artificial bias is introduced into LaDyBUGS free energies as a result of using dynamic bias updates.

### Symmetric methyl perturbations

Symmetric perturbations were performed with LaDyBUGS to interconvert between identical but distinct methyl groups on toluene and p-xylene (as shown in Supplementary Fig. 1). This test confirms that correct sampling and accurate free energy estimates could be obtained without introducing artifacts via continuous bias updates in LaDyBUGS. As shown in Table 1, for both systems, the expected result (Δ*G* = 0.00 kcal mol^−1^) is reproduced within the computed bootstrapped errors, suggesting that LaDyBUGS is functioning properly for both single-site (toluene) and multisite (p-xylene) systems. These results provide confidence to proceed with benchmark perturbations commonly observed in structure-based drug design.

**Table 1:** Free energy differences in water for symmetric methyl perturbations computed with LaDyBUGS (kcal mol^−1^)

Site 1 (X)	Site 2 (Y)	Δ*G*_water_ ± *σ*_*M*_
**Toluene → Toluene**		
CH_3_ (A)	H	0.00
CH_3_ (B)	H	0.0018 ± 0.004
**p-Xylene → p-Xylene**		
CH_3_ (A)	CH_3_ (C)	0.00
CH_3_ (A)	CH_3_ (D)	0.0005 ± 0.003
CH_3_ (B)	CH_3_ (C)	0.0003 ± 0.004
CH_3_ (B)	CH_3_ (D)	−0.0015 ± 0.004

Pairs of symmetric but distinct CH_3_ groups are labeled (A, B) and (C, D). Toluene and p-xylene structures are shown in Supplementary Fig. 1. Errors are presented as the standard error of the mean (*σ*_*M*_).

### Structure-based drug design benchmarking

**Fig. 4:**
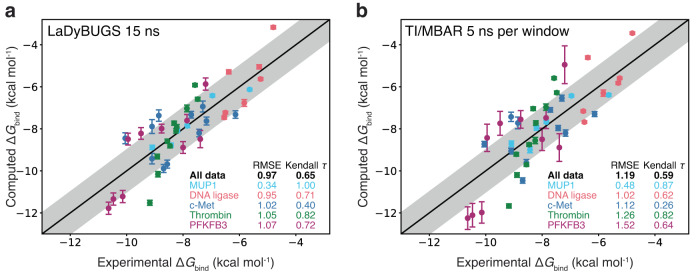
Correlation to experiment. Computed **a** LaDyBUGS 15 ns and **b** TI/MBAR 5 ns per window Δ*G*_bind_ compared to experiment (in kcal mol^−1^). Data points are colored by the chemical system (MUP1 in cyan, DNA ligase in red, c-Met in blue, thrombin in green, and PFKFB3 in purple), and bootstrapped uncertainties computed over three replicates with FastMBAR are shown as error bars. The center black line represents perfect one-to-one correlation; the shaded gray area represents an error of ± 1 kcal mol^−1^. Root-mean-square errors and Kendall *τ* statistics are reported for all data points collectively and each system individually. Source data are provided as a Source Data file.

**Fig. 5:**
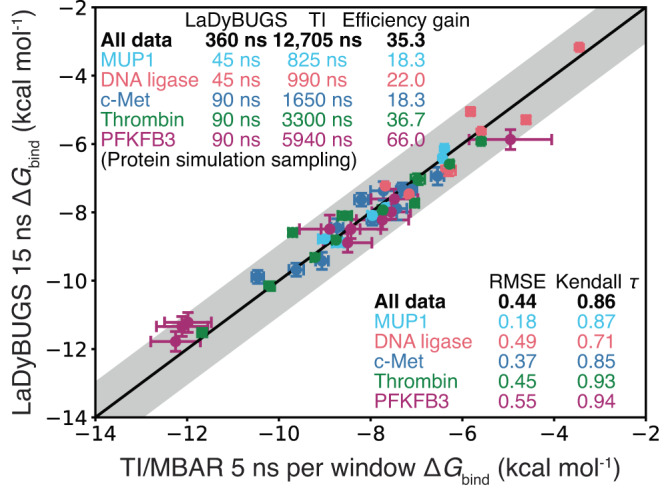
Correlation to TI/MBAR. Correlation between computed LaDyBUGS 15 ns and TI/MBAR 5 ns per window Δ*G*_bind_ results (in kcal mol^−1^). Data points are colored by chemical system (MUP1 in cyan, DNA ligase in red, c-Met in blue, thrombin in green, and PFKFB3 in purple), and bootstrapped uncertainties computed over three replicates with FastMBAR are shown as error bars. The center black line represents $y=x$; the shaded gray area represents an error of ±1 kcal mol^−1^. Root-mean-square errors, Kendall *τ* statistics, total amount of sampling, and efficiency gains of LaDyBUGS 15 ns over TI/MBAR 5 ns per window in terms of sampling are reported. Source data are provided as a Source Data file.

To demonstrate the applicability of LaDyBUGS for structure-based drug design, we assess the accuracy and efficiency of the LaDyBUGS method compared to experiment and a standard alchemical free energy method (TI/MBAR). We also briefly compare LaDyBUGS vs *λ*D, since both methods can examine multiple perturbations simultaneously, albeit *λ*D requires additional sampling time to identify biases prior to production sampling. In total, free energies of binding were calculated for 45 ligands bound to one of five benchmark protein systems: MUP1, DNA ligase, c-Met, thrombin, or PFKFB3. Figure 4 plots the correlation between the experiment and computed binding affinities (Δ*G*_bind_) with LaDyBUGS (using 15 ns of sampling per simulation) and TI/MBAR (using 5 ns of sampling per *λ* window; 55 ns of total sampling per pairwise perturbation); all data points are reported in Supplementary Tables 1 and 2 of the Supplementary Information. Root-mean-square error (RMSE) and Kendall *τ* scores^59^ were computed for each test system individually and for the combined dataset^60^. Using these metrics, we see a uniform improvement in both RMSE and Kendall *τ* with LaDyBUGS relative to TI/MBAR. For all 45 ligands, the LaDyBUGS RMSE was 0.97 kcal mol^−1^ and the Kendall *τ* was 0.65. For every test case, the calculated LaDyBUGS RMSE was near or below 1.0 kcal mol^−1^, a typical goal and state-of-the-art for predictive accuracy in free energy calculations for drug discovery^5–7,11,16,17,24^. It is important to note that accuracy is dependent on both correct force field representation of a chemical system and thorough configurational sampling with a given free energy method^61^. The larger RMSE of 1.19 kcal mol^−1^ and reduced Kendall *τ* of 0.59 from TI/MBAR (5 ns per window), which used the same force field parameters as LaDyBUGS, suggests LaDyBUGS is providing improved sampling proficiency over TI/MBAR for the same benchmark systems. Notably, LaDyBUGS used 18.3–66.0 times less sampling than TI/MBAR 5 ns per window when comparing the two computational approaches (Fig. 5).

**Fig. 6:**
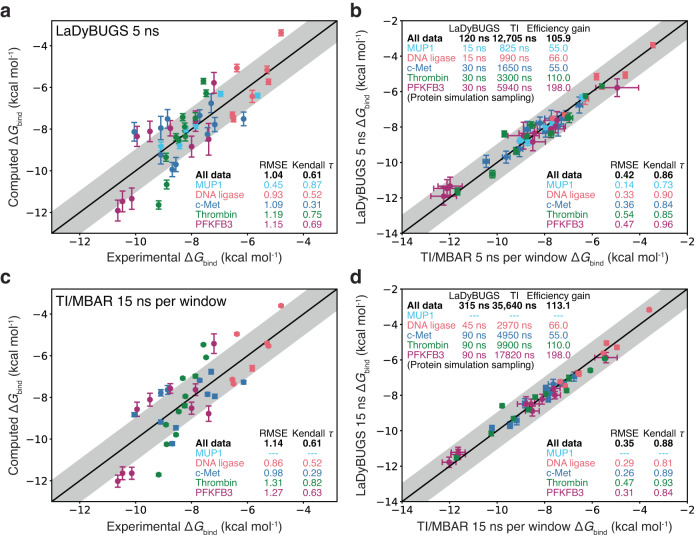
Correlation associated with changes in sampling. Correlation in Δ*G*_bind_ between **a** LaDyBUGS 5 ns compared to experiment, **b** LaDyBUGS 5 ns compared to TI/MBAR 5 ns per window, **c** TI/MBAR 15 ns per window compared to experiment, and **d** LaDyBUGS 15 ns compared to TI/MBAR 15 ns per window. Data points are colored by chemical system (MUP1 in cyan, DNA ligase in red, c-Met in blue, thrombin in green, and PFKFB3 in purple), and bootstrapped uncertainties computed over three replicates with FastMBAR are shown as error bars. The center black line represents ideal one-to-one agreement; the shaded gray area represents an error of ± 1 kcal mol^−1^. Root-mean-square errors, Kendall *τ* statistics, total amount of sampling, and efficiency gains of LaDyBUGS over TI/MBAR in terms of sampling are reported. Source data are provided as a Source Data file.

Because both LaDyBUGS and TI/MBAR calculations used the same force field parameters, we can also compare the agreement of their Δ*G*_bind_ predictions (Fig. 5). The two computational methods agree well with each other, with an overall RMSE of 0.44 kcal mol^−1^. Considering that most protein–ligand Δ*G*_bind_ calculations have computed uncertainties between 0.3–0.5 kcal mol^−1^ ^16,48,49^, and that LaDyBUGS bootstrapped errors ranged from 0.1 to 0.4 kcal mol^−1^ (Supplementary Tables 1 and 2), these results suggest good agreement between these free energy methods exists and that LaDyBUGS results are comparable to the community accepted standard TI/MBAR. To explore the effect of sampling time on the Δ*G*_bind_ results, we also compared these methods with less sampling per LaDyBUGS simulation (with 5 ns of sampling per simulation) and more sampling per TI/MBAR calculation (with 15 ns of sampling per *λ* window; 165 ns of total sampling per pairwise perturbation). In Fig. 6, the agreement between LaDyBUGS 5 ns simulations compared to TI/MBAR 5 ns per window remains high with a RMSE of 0.42 kcal mol^-1^ and a Kendall *τ* of 0.86. As expected with a reduction in sampling, the mean bootstrapping error for LaDyBUGS increased from 0.17 to 0.30 kcal mol^−1^, although the RMSE between LaDyBUGS (5 ns) and experiment remains comparable at 1.04 kcal mol^−1^. This level of agreement is significant considering LaDyBUGS 5 ns used 55.0–198.0 times less sampling than TI/MBAR 5 ns per window. The amount of TI sampling for thrombin and PFKFB3 are noticeably larger than the other systems, by a factor of 2–4, due to the additional calculations performed to sample alternate conformations of larger aromatic ring perturbations. As discussed in the next subsection, LaDyBUGS showed enhanced sampling of dihedral torsions for alchemical aromatic rings, while TI/MBAR did not, requiring additional sampling to be manually performed. Thus, from a total of 120 ns expended to sample all 45 ligands (~2.67 ns per ligand) bound to their respective targets, LaDyBUGS 5 ns can provide Δ*G*_bind_ predictions with errors near or below 1.0 kcal mol^−1^ compared to the experiment. Though error bars are slightly larger than observed with 15 ns, running LaDyBUGS for 5 ns could provide a useful way to quickly screen large series of ligand analogs prior to more rigorous evaluations by extending sampling to longer time scales. These results further highlight that significant cost savings are achievable with LaDyBUGS without compromising accuracy in the computed Δ*G*_bind_ results. In contrast, with 5 ns of sampling per window, TI/MBAR required 12.7 μs of total protein–ligand sampling (165–660 ns per ligand). We note that commonly employed redundant calculations for cycle closure and hysteresis error reduction were not performed here to try to maximize the efficiency of TI/MBAR, although additional calculations were performed to sample 180° rotated ring conformations of the thrombin and PFKFB3 alchemical substituents^18–20^. To test LaDyBUGS convergence, 25 ns simulations were also run. No large deviations were observed and the RMSE between LaDyBUGS 15 ns and LaDyBUGS 25 ns simulations was small (0.12 kcal mol^−1^), well within statistical noise (Supplementary Tables 1 and 2). The RMSE of 0.30 kcal mol^−1^ between 5 ns and 25 ns LaDyBUGS results was slightly larger but still within noise, suggesting a high degree of convergence even with a minimal amount of LaDyBUGS sampling. Figure 6 also shows the effects of extending TI/MBAR sampling to 15 ns per window for all systems except MUP1, which was deemed to be satisfactorily converged at 5 ns per window by its low RMSE compared to LaDyBUGS. Large improvements are observed for TI/MBAR 15 ns per window in comparison to TI/MBAR results from 5 ns per window of sampling. The RMSE to experiment improves to 0.93 kcal mol^−1^ with TI/MBAR 15 ns per window, and the RMSE to LaDyBUGS decreases to 0.27 kcal mol^−1^. The strong agreement between short 5 ns runs of TI/MBAR and LaDyBUGS, as well as between the longer 15 ns runs of TI/MBAR and LaDyBUGS, suggests LaDyBUGS is able of deliver comparable accuracy as TI/MBAR with significant cost savings in terms of sampling (18–200 times less simulation time required). This is notable considering the spectrum of alchemical substituent sizes involved in these benchmark systems: from 1 to 4 heavy atoms in MUP1 and DNA ligase systems to 6–12 heavy atoms and entire aromatic rings in c-Met, thrombin, and PFKFB3. Improved efficiency with LaDyBUGS directly stems from its ability to investigate several alchemical perturbations collectively within a single simulation, without the need to break up transformations into separate *λ* windows spread across multiple separate simulations (Fig. 2).

The performance of LaDyBUGS can also be compared to *λ*D since both methods are able to sample multiple substituent transformations simultaneously. As shown in Supplementary Fig. 2, high correlation is observed between these *λ*D-based techniques; the RMSE is 0.45 kcal mol^−1^ and the Kendall *τ* is 0.83. Overall, these results are very similar to what was observed in the above comparisons of LaDyBUGS and TI/MBAR. In contrast to LaDyBUGS, however, *λ*D required 609 ns of sampling for bias identification prior to its 360 ns of production sampling. At this level of sampling, *λ*D is still 13.1 times more efficient than TI/MBAR 5 ns per window, but 2.7 times less efficient than LaDyBUGS 15 ns. The loss of efficiency of *λ*D compared to LaDyBUGS stems directly from the costs associated with bias identification for *λ*D. Each system used a minimum of 48 ns for initial bias identification; however, additional production runs were performed for DNA ligase and thrombin because initial biases were not converged, causing poor *λ* sampling and free energy convergence in initial production calculations. Though biases can be readily refined with ALF, production sampling that isn’t used for production results ultimately gets incorporated into a system’s overall bias identification costs. For example, in initial DNA ligase production simulations, the reference compound was sampled less than 1% of the time in two out of three duplicates, yielding high uncertainties in computed Δ*G*_bind_. Similar trends were observed for some thrombin molecules as well, which were sampled only 2–4% of the time. Meanwhile, other ligands predominated and were sampled 40–50% of the time. These deficiencies were mostly resolved after refining biases and rerunning production simulations, although DNA ligase *λ*D results still show some larger uncertainties (>0.5 kcal mol^−1^) and may benefit from additional sampling and bias optimization. Thrombin *λ*D results appeared well converged and ligand end states were more equally sampled in the final production runs. As discussed in more detail below, LaDyBUGS avoids these problems of both bias identification and uneven *λ* sampling via the use of dynamic bias updates. This allows LaDyBUGS to be efficiently and accurately run with less sampling (5–15 ns). In contrast, shortening bias flattening in *λ*D would yield poorer performance because biases may not be fully optimized. As an example, LaDyBUGS 5 ns yields a consistent RMSE of 0.41 kcal mol^−1^ compared to *λ*D but is now 8.1 times more efficient. In summary, high cost savings are observed with LaDyBUGS even when its performance is compared to other expanded ensemble techniques that can similarly examine multiple perturbations simultaneously.

### Enhanced sampling of dihedral torsions with LaDyBUGS

In addition to the efficiency improvements observed with LaDyBUGS, enhanced sampling of dihedral torsions for large substituent perturbations was also observed with LaDyBUGS. In fact, this behavior has long been observed with *λ*D, where intramolecular degrees of freedom in alchemical substituents can be scaled by *λ* at a user’s discretion. To preserve a functional group’s expected geometric shape, dihedral angles are often scaled by *λ*, but bonds and angles are not^42,43,49^. Furthermore, by sampling multiple substituents simultaneously, *λ*D-based methods provide greater flexibility and time in the MD simulation for substituents to sample alternative conformations when their respective *λ* states are near 0. These attributes are retained in LaDyBUGS, and they provide enhanced sampling for perturbations of larger functional groups, such as the aromatic ring transformations in thrombin and PFKFB3 systems. As shown in Supplementary Fig. 3, LaDyBUGS can equally sample two flipped ring conformations for both thrombin and PFKFB3 ligands within a single calculation, while TI/MBAR is clearly trapped in a single starting conformation. Direct comparison of TI/MBAR results without considering ring flips would, thus, be expected to yield poor agreement to LaDyBUGS, because TI/MBAR fails to sample these important conformational changes. For example, without considering ring flips in the TI/MBAR results, RMSEs of 0.68 and 1.01 kcal mol^-1^ are observed when comparing LaDyBUGS 15 ns vs TI/MBAR 5 ns per window for thrombin and PFKFB3 systems, respectively. These RMSEs drop significantly to 0.45 and 0.55 kcal mol^−1^ (Fig. 5), respectively, when additional TI/MBAR calculations with rotated functional groups are performed and weighted together with MBAR to calculate the final Δ*G*_bind_ results. Hence, LaDyBUGS can provide enhanced sampling of large substituents, without incorporating replica exchange or other additional enhanced sampling techniques, allowing it to retain a high degree of efficiency and speed for examining many kinds of different alchemical perturbations.

### Uniformity of *λ* sampling with LaDyBUGS

**Fig. 7:**
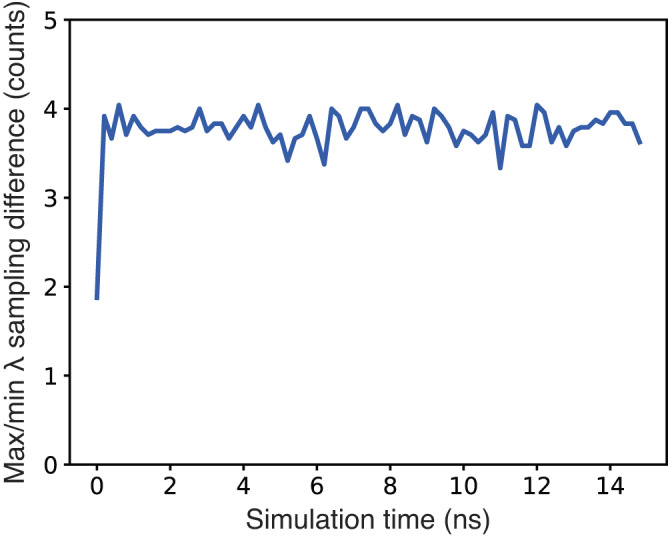
Sampling counts. The average difference between the maximum and minimum number of times a *λ* state was sampled. Counts were computed from all LaDyBUGS benchmark simulations and averaged together as a function of time (plotted in blue). Source data are provided as a Source Data file.

One potential issue associated with expanded ensemble free energy methods is a difficulty in achieving sampling smoothness of all *λ* states, e.g., avoiding becoming stuck primarily sampling one or several states too often and neglecting to sample all other *λ* states^41,62^. This problem has been observed in a recent expanded ensemble investigation that used a Wang–Landau algorithm to propagate *λ* switching^28^. In conventional *λ*D simulations, including MSλD and d-GSλD, static biases are added to a simulation to reduce free energy barriers in *λ* space and facilitate transitions between *λ* states^41,47^. In most situations, these biases work well, all *λ* states are evenly sampled, and reliable free energy predictions are obtained. But, as discussed above for *λ*D, burn-in time is required to first identify appropriate biases for these methods, which decreases their overall efficiency. Furthermore, if biases are poorly converged, static biases may be unable to facilitate continuous *λ* sampling and the simulation could become trapped sampling one or a handful of alchemical end states. Thus, new biases would need to be identified and sampling would have to be restarted. This was observed in the present *λ*D work with DNA ligase and thrombin benchmark systems. However, the dynamic biases used in LaDyBUGS continuously propagate the sampling of many *λ* states without prior burn-in time for bias identification and allow for conformational plasticity of the chemical system without getting trapped sampling a small number of *λ* states. Biases from Eq. (4), in “Methods”, rely solely on the number of times each *λ* state has been sampled and on-the-fly FastMBAR free energy estimates; thus, LaDyBUGS can provide incredibly smooth *λ* sampling throughout an entire simulation. Figure 7 shows the difference between the minimum and maximum number of times a *λ* state was sampled as a function of time, referred to as “counts”, averaged across all protein simulations used for benchmarking. On average, the difference between minimum and maximum counts is ~4, even though *λ* states are sampled more than 500–800 times by the end of each simulation. This level of sampling smoothness ensures that LaDyBUGS does not become trapped sampling particular *λ* states and provides rapid transitions between multiple ligand end states to facilitate accurate free energy estimation with FastMBAR.

### LaDyBUGS samples a mixture distribution of *λ* states

**Fig. 8:**
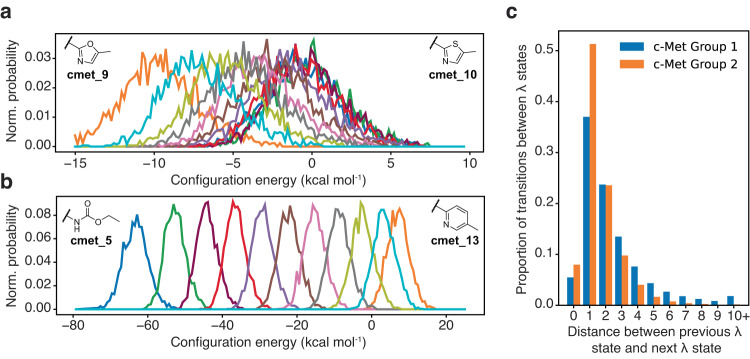
Analysis of *λ* state transition probabilities. Normalized configuration energy distributions between adjacent *λ* states in two example LaDyBUGS perturbations: **a** c-Met group 1 cmet_9 → cmet_10 and **b** c-Met group 2 cmet_5 → cmet_13. Each colored line represents a distribution associated with a unique *λ* state progressing from *λ* = 0.0 on the left to *λ* = 1.0 on the right with Δ*λ* steps of 0.1 (*λ* = {0.0, 0.1, 0.2, …, 0.9, 1.0}). **c** Normalized probabilities of transition distances between *λ* states sampled in all c-Met group 1 and c-Met group 2 LaDyBUGS simulations. The greater the degree of overlap between adjacent *λ* states, the more probable long-range *λ* transitions become. Additional transition probabilities can be found in Supplementary Table 3. Source data are provided as a Source Data file.

Smooth transitions between states are also facilitated by strong energetic overlap between neighboring *λ* states. In our benchmark studies, c-Met group 1 consists of different 5-membered heterocycles while group 2 contains a mixture of carbamate and aryl substituents (Fig. 3). As shown in Fig. 8 for two example c-Met group 1 and 2 perturbations, a uniform Δ*λ* schedule provides good energetic overlap between both similar (c-Met Group 1) and dissimilar (c-Met Group 2) transformations. This enables facile transitions to adjacent *λ* states when sampling the *P*(*λ* | *X*) conditional distribution (described more in “Methods”). As shown in Fig. 8, most transitions occur to +1 or +2 states away, although large jumps (>4 states) are sometimes observed. The degree of overlap between *λ* states affects the transition distance traveled, with higher overlap facilitating larger jumps (see also Supplementary Table 3). The mean transition distance traveled for c-Met group 1 is 2.47 states, but it is smaller at 1.64 states for c-Met group 2 which has less overlap between adjacent states (Fig. 8). Fortuitously, transitions between energetically similar and adjacent *λ* states enables the chemical system to quickly relax and equilibrate during the brief 200 fs MD simulation following a sampling transition to a new *λ* state. Therefore, we assume the MD configuration drawn from *P*(*X* | *λ*) in each Gibbs sampling step represents an equilibrium sample. By constant sampling of different *λ* states and atomic coordinates, LaDyBUGS can efficiently sample a mixture distribution of *λ* states within a single simulation. Pairing free energy determination with the MBAR algorithm is natural then, because MBAR pools and reweights samples as if they originated from a mixture distribution^15,63^. Supplementary Note 1 presents a mathematical proof that demonstrates that samples drawn from the same *λ* state with different external biases can be treated as coming from the same state. We then use FastMBAR to obtain equilibrium free energy results from a LaDyBUGS simulation, under the stated assumptions of the proof. Furthermore, because sampling of the *P*(*λ* | *X*) conditional distribution requires energies to be calculated for every *λ* state at every sampled *P*(*X* | *λ*) configuration, no postprocessing of LaDyBUGS trajectories is required to run MBAR; all necessary information is generated on-the-fly and is available at the conclusion of a LaDyBUGS simulation.

### Software implementation

LaDyBUGS has been implemented in OpenMM^51^, and all LaDyBUGS scripts are available for download on the Vilseck Lab GitHub page. One advantage of using OpenMM for LaDyBUGS is the ability to use force groups to partition the interactions of different components of an alchemical system and thus enable *λ* state-dependent energies to be evaluated without recalculating the energy of the entire chemical system. This feature speeds up the sampling of *P*(*λ* | *X*) which requires *λ*-dependent energies to be calculated for every *λ*-state at every *P*(*X* | *λ*) configuration. Consequently, we find that sampling a group of 6 ligands collectively with 141 *λ* states is only marginally slower than performing a standard pairwise perturbation of 11 *λ* states with LaDyBUGS. For example, on a NVIDIA 2080 TI GPU, 6 duplicate 5 ns LaDyBUGS c-Met group 1 cmet_9 to cmet_10 pairwise perturbations each took ca. 10.85 h to run. Similarly, 6 duplicate 5 ns simulations of all 6 c-Met group-1 ligands sampled collectively took ca. 11.01 h each. Thus, the combined 6-ligand calculation was only ca. 1.5% slower, highlighting the effectiveness and cost savings of sampling multiple ligands simultaneously with LaDyBUGS. With our current implementation of LaDyBUGS in OpenMM and using an assumption of sampling 6 perturbations per LaDyBUGS simulation, we estimate that ca. 4–13 compound perturbations can be investigated per day per 1 GPU with LaDyBUGS using a range of 15 ns to 5 ns of sampling per calculation, respectively. On a modest cluster of 25 GPUs, this readily scales to 100-325 perturbations per day. Hence, rapid high-throughput screening of hundreds of lead compound analogs with highly accurate free energy predictions is obtainable with LaDyBUGS within a day using minimal computational resources.

Work is ongoing to further optimize our implementation of LaDyBUGS in OpenMM as well as incorporate it into other software suites, including CHARMM. To date, LaDyBUGS has been implemented in pyCHARMM, a python API for CHARMM^64^. In these efforts, if a program lacks the ability to partition energetic interactions via a “force group”-like algorithm, *P*(*λ* | *X*) may be sampled by calculating the energy of the entire chemical system; all non-alchemical environment-to-environment interactions should cancel out when *λ* state-dependent energies are compared. Though some wall-time slowdown may be expected to occur as a consequence of running a larger energy evaluation, we anticipate that LaDyBUGS would still provide highly efficient results, nonetheless. Incorporating LaDyBUGS into CHARMM, or other programs, could provide additional benefits too. For example, the CustomNonbondedForce class in OpenMM makes it challenging to use particle mesh Ewald (PME) methods with LaDyBUGS. However, a *λ*D-based PME approach is already available in CHARMM and BLaDE for running *λ*D simulations^45,65–67^, and this can be utilized with LaDyBUGS in pyCHARMM to facilitate the inclusion of long-range electrostatic interactions in future calculations.

### Multisite sampling

Finally, we emphasize that the efficiency gains for LaDyBUGS reported in this work used only single-site perturbations, where substituent group modifications occurred at only one site off a central ligand core. Multisite perturbations, with functional group substitutions occurring at multiple sites around a ligand core, could also be accomplished as performed previously with d-GSλD^47^. Such LaDyBUGS simulations may need longer total sampling to obtain converged results due to the increased number of *λ* states required for multisite sampling, but this has not yet been tested. Instead, this work focused on single-site perturbations to match structure-activity relationship strategies typically pursued experimentally by changing one component of a lead compound at a time^16,52–56^. In this manner, LaDyBUGS seems especially adept at exploring incremental changes to a lead compound. Future investigations will reveal the applicability of LaDyBUGS to tackle larger or more challenging perturbations or for molecular decoupling to compute absolute free energies of binding directly.

### Summary and outlook

Alchemical free energy methods such as FEP and TI have played pivotal roles in the lead optimization phase of drug design^1–3,5–11^, yet they require large computational costs to explore many tens to hundreds of alchemical perturbations. *λ*D-based methods have shown improved scalability and efficiency in exploring large chemical spaces with reduced costs^6,33,34,44,46–49^. Hence, the object of this study was to investigate approaches to further accelerate *λ*D-based free energy methods by eliminating burn-in time commonly expended to identify static biases prior to production sampling. In this work, we have described the *λ*-dynamics with bias-updated Gibbs sampling method, which is a Gibbs sampler-based *λ*-dynamics approach. To eliminate time spent for bias identification, LaDyBUGS uses continuous bias updating to rigorously drive the sampling of multiple *λ* states, and consequently multiple different ligands, simultaneously within a single simulation. This results in very even and complete sampling of all *λ* states and significant efficiency gains, compared to TI/MBAR. Evaluated against five experimental benchmarks, LaDyBUGS RMSEs of computed Δ*G*_bind_ compared to the experiment were less than 1 kcal mol^−1^ on average with only 5–15 ns of sampling per simulation. LaDyBUGS RMSEs were lower than the corresponding error with TI/MBAR in all test cases, notwithstanding the use of only ca. 2–5% of the total amount of TI sampling. From these results, we estimate that highly accurate Δ*G*_bind_ estimates can be obtained with only ca. 2.5–5 ns of LaDyBUGS sampling per ligand. From timing benchmarks of LaDyBUGS implemented in OpenMM, we estimate that ca. 4–13 perturbations can be examined per day per GPU with LaDyBUGS, depending on the length of sampling. Using a modest amount of GPU resources (with as few as 25 GPUs), this can easily scale to hundreds of compounds examined within a day. We envision that the rapid delivery of Δ*G*_bind_ predictions via LaDyBUGS could thus be used to screen hundreds of compound analogs with minimal computational costs, accelerating computer-aided drug discovery at an incredible pace.

## Methods

*λ*-Dynamics with bias-updated Gibbs sampling builds upon the framework of the discrete Gibbs sampler *λ*-dynamics approach^47^. Therefore, we quickly review d-GSλD before describing the workflow for LaDyBUGS.

### Discrete Gibbs sampler *λ*-dynamics

To investigate alchemical transformations of a chemical system, d-GSλD samples the joint distribution of atomic coordinates, *X*, and alchemical states, *λ*, (*P*(*X*, *λ*))^47^. With Gibbs sampling this is accomplished via indirect sampling of two related conditional distributions, *P*(*X* | *λ*) and *P*(*λ* | *X*), which are formed by freezing a subset of variables, *λ* and *X,* respectively. A single Gibbs sampler step thus consists of sequential sampling of *P*(*X* | *λ*) and *P*(*λ* | *X*) to yield *X* and *λ* at time *t* (*X*_*t*_, λ_*t*_)^32,68,69^. To obtain *X*_*t+1*_, molecular dynamics can be used to sample *P*(*X*_*t*_ | λ_*t*_), the coordinate space of the chemical system; *λ*_*t*+1_ can then be chosen by using a pseudorandom number generator to sample *P*(*λ*_*t*_ | *X*_*t*+1_) (described in more detail below). While GSλD can utilize both continuous and discrete *λ* variables, the use of discrete *λ* states in d-GSλD was advantageous for several reasons. Notably, it allowed for soft-core potentials, or other *λ*-dependent potentials, to be easily integrated into the direct sampling routines of *P*(*λ* | *X*); in contrast, with the continuous *λ* variant of GSλD, use of a nonlinear *λ*-dependent potential creates a complex normalization constant in *P*(*λ* | *X*) which prevents direct sampling^46,47^. Also, d-GSλD facilitated the exploration of multiple perturbations at many sites around a central ligand core^47^. Though no unique solution exists for defining discrete *λ* states between multiple ligand end states, a representation of *λ* states along connective edges between ligands provides a strongly connected map for sampling multiple ligands simultaneously (Fig. 2), and it has yielded good free energy results in prior benchmark evaluations^47^. We note that to sample multiple ligands simultaneously with d-GSλD, a single *λ* state *i* (*λ*^*i*^) consists of a vector of substituent-specific *λ*_*y,c*_ variables (for substituent *c* at site *y*) that scale the interactions of each alchemical functional group individually^47^. Like most *λ*D-based methods, all *λ*_*y,c*_ values within a single *λ*^*i*^ state must sum to 1.0 to prevent more than 1 ligand from interacting with the rest of the chemical system at one time^34,41,42^. Furthermore, as mentioned earlier, biases are necessary to reduce free energy barriers in *λ* space and facilitate transitions between *λ*^*i*^ states at equilibrium. For d-GSλD, these biases are a single scalar energy term added to each *λ*^*i*^ state^47^. Prior to production sampling, static biases for each *λ*^*i*^ state were identified with a Wang–Landau-like algorithm with ca. 5–10 ns of sampling^70,71^. Production sampling for a preset amount of Gibbs sampling steps then ensued, followed by a FastMBAR^50^ calculation to compute all final relative free energy differences.

### *λ*-Dynamics with bias-updated Gibbs sampling

**Fig. 9:**
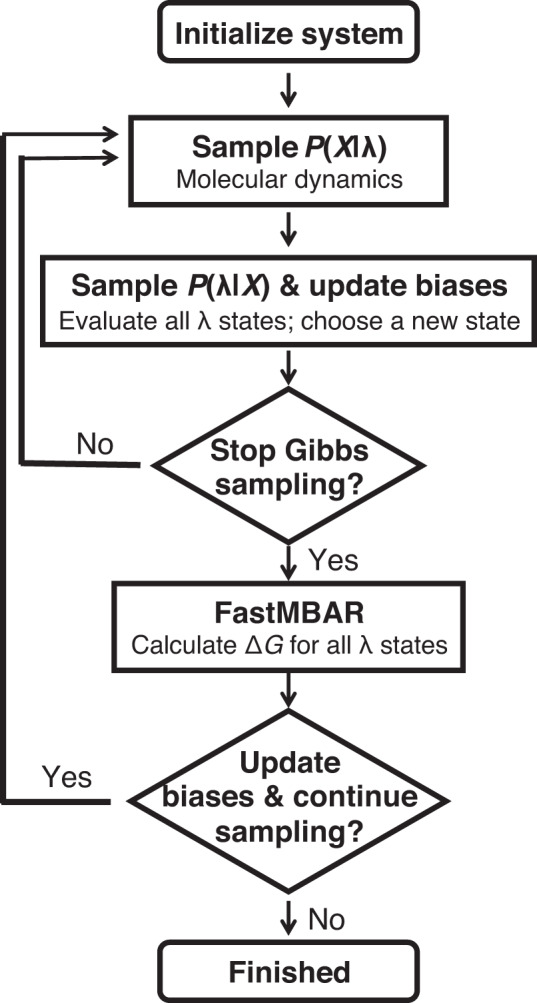
The LaDyBUGS workflow. Gibbs sampling is used to sample atomic coordinates and *λ* states of an alchemical system; a dynamic bias is continually updated to ensure continuous sampling of all *λ* states. Final free energy estimates and periodic bias refinements are accomplished with FastMBAR.

LaDyBUGS builds upon the d-GSλD framework and uses Gibbs sampling with discrete *λ* states to sample multiple ligand end states collectively within a single simulation. However, in an endeavor to accelerate d-GSλD and achieve rapid free energy results, Gibbs sampling is performed with dynamic biases, rather than static biases, to drive the exploration of many *λ* states during production sampling without prior bias determination. Figure 9 describes the workflow of LaDyBUGS. Following initialization and minimization of a chemical system, the atomic coordinates and alchemical states of the system are alternatively sampled with Gibbs sampling. As described above, *P*(*X* | *λ)* can be sampled with MD. Like in d-GSλD, in LaDyBUGS the conditional distribution *P*(*λ* | *X*) can be described as a multinomial distribution (Eq. (1)):

1 $$P\left({{{{{{\rm{\lambda }}}}}}}^{i}\big|X\right)=\frac{\exp \left(-\beta \left[{V}_{{{{{{\rm{SS}}}}}}}\left(X,\, {{{{{{\rm{\lambda }}}}}}}^{i}\right)+{V}_{{{{{{\rm{MS}}}}}}}\left(X,\, {{{{{{\rm{\lambda }}}}}}}^{i}\right)+{E}^{i}\right]\right)\,}{{\sum }_{l=1}^{M}\exp \left(-\beta \left[{V}_{{{{{{\rm{SS}}}}}}}\left(X,\, {{{{{{\rm{\lambda }}}}}}}^{l}\right)+{V}_{{{{{{\rm{MS}}}}}}}\left(X,\, {{{{{{\rm{\lambda }}}}}}}^{l}\right)+{E}^{l}\right]\right)\,}$$

where *M* represents the total number of *λ*^*i*^ states and *E*^*i*^ is a scalar bias added to each *λ*^*i*^ state. The single-site *V*_SS_ and multisite *V*_MS_ potentials, necessary for investigating multisite perturbations of substituents *c* and *d* at sites *y* and *z*, are defined by Eqs. (2) and (3), respectively:

2 $${V}_{{{{{{\rm{SS}}}}}}}\big(X=\big({x}_{0},\, \{x\}\big),\, {{{{{\rm{\lambda }}}}}}\big)=\mathop{\sum }\limits_{y=1}^{S}\mathop{\sum }\limits_{c=1}^{{N}_{y}}{{{{{{\rm{\lambda }}}}}}}_{y,c}\left(V\left({x}_{0},\, {x}_{y,c}\right)+V\left({x}_{y,c}\right)\right)$$

3 $${V}_{{{{{{\rm{MS}}}}}}}(X=({x}_{0},\, \{x\}),\, {{{{{\rm{\lambda }}}}}})=\mathop{\sum }\limits_{y=1}^{S}\mathop{\sum }\limits_{c=1}^{{N}_{y}}\mathop{\sum }\limits_{z=y+1}^{S}\mathop{\sum }\limits_{d=1}^{{N}_{z}}{{{{{{\rm{\lambda }}}}}}}_{y,c}{{{{{{\rm{\lambda }}}}}}}_{z,d}V({x}_{y,c},\, {x}_{z,d})$$

where *X* comprises atomic coordinates for both environment (*x*_*0*_) and alchemical components (*x*_*y,c*_), {*x*} represents the set of all *x*_*y,c*_ coordinates, *S* represents the number of sites, *N*_*y*_ is the number of substituents on-site *y*, and *λ*_*y,c*_ are the site- and substituent-specific *λ* variables. These equations stem from similar potentials used in conventional MSλD for multisite sampling^34,41–47^. However, if systems with only single-site modifications are investigated, *V*_MS_ equals zero and can be ignored. The conditional distribution *P*(*λ* | *X*) at time *t* is thus formed by first calculating the potential energy of the system at coordinates *X*_*t*_ for each alchemical state *λ*^*i*^ and normalizing to form a Boltzmann distribution. A new *λ*^*i*^ at time *t* + *1* (${{{{{{\rm{\lambda }}}}}}}_{t+1}^{i}$) state can then be chosen by selecting a new state proportional to its probability with a pseudorandom number generator. As shown in Fig. 9, Gibbs sampling is an iterative process that is performed repeatedly for a preset amount time, usually quantified as an amount of cumulative MD sampling. Prior to the end of each Gibbs sampler step, the biases for all *λ*^*i*^ states are also updated. This update step is described in more detail in the next section. Furthermore, at designated break points, Gibbs sampling is halted and FastMBAR is called to compute relative free energy differences (Δ*G*^*i*^) for each *λ*^*i*^ state compared to the reference state, *λ*^1^. These intermediate MBAR free energy results, collected at various stages of an ongoing simulation, can be used to update and refine the *E*^*i*^ biases for the next set of Gibbs sampler steps. As discussed below, this helps provide uniform sampling of all *λ* states in a LaDyBUGS simulation. Advantageously, the MBAR input, i.e., equilibrium energies of all *λ* states at every sampled configuration of the system (*X*), are calculated and saved on-the-fly when *P*(*λ* | *X*) is sampled, thus no trajectory postprocessing is necessary to combine MBAR with LaDyBUGS. Finally, a concluding MBAR calculation is performed at the termination of a LaDyBUGS free energy calculation to compute the final relative free energy results.

### Choice of the bias function

In LaDyBUGS, the *E*^*i*^ biases are changed at the end of each Gibbs sampler step and intermediate FastMBAR calculations are performed regularly throughout a simulation to provide additional bias refinement. When a LaDyBUGS simulation is initiated, a relatively aggressive biasing scheme is used to ensure every *λ* state is sampled prior to running FastMBAR for the first time. For example, in this work we used a flat external bias of 100 kcal mol^−1^, which is added to each *λ*^*i*^ state every time that state is sampled. While any flat bias value would work, in principle, a large bias (≥ 10 kcal mol^−1^) ensures rapid sampling of all *λ* states within a small amount of Gibbs sampling near the onset of a LaDyBUGS simulation. Prior to the first iteration of running FastMBAR, the total bias on *λ*^*i*^ is ${E}^{i}=100\,{L}_{i}$, where *L*_*i*_ is the number of times *λ*^*i*^ was sampled and 100 is the flat bias employed in this work. At time *t* = *u* updates, Gibbs sampling is stopped and a FastMBAR calculation is performed to estimate the free energy differences of each *λ*^*i*^ state up to that point in time ($\Delta {G}_{t=u}^{i}$). At this stage, the *E*^*i*^ biases are replaced with the negative value of the MBAR results ($-\Delta {G}_{t=u}^{i}$) and an additional exponential bias^39^ is used to penalize each *λ*^*i*^ state based on the number of times *λ*^*i*^ is sampled compared to the least-sampled state (min[*L*(*λ*)]) (Eq. (4), where ε_*b*_ = 1.0 kcal mol^−1^). After each Gibbs sampler step, the biases are updated with Eq. (4) to reflect the new number of counts per *λ*^*i*^ state, but the $-\Delta {G}_{t=u}^{i}$ component remains unchanged until the next FastMBAR calculation. Through this continuous changing of the biases, complete and smooth sampling of all *λ* states can be achieved (see “Results and discussion” and Fig. 7). Supplementary Note 1 presents a mathematical proof that, assuming the MD simulation used for sampling from *P*(*X* | *λ*) in each Gibbs sampler step reaches equilibrium, the value of the scalar bias used during Gibbs sampling has no effect on the FastMBAR calculation, facilitating the use of unbiased equilibrium energies in FastMBAR for free energy estimation.

4 $${E}^{i}=-\varDelta {G}_{t=u}^{i}+{\varepsilon }_{b}{2}^{{L}_{i}-\min [L(\lambda )]}$$

### Benchmark system details

LaDyBUGS has been implemented in OpenMM and all simulations were run using the CUDA platform^51^. CHARMM-based force field parameters were used to represent different components of the chemical systems. CHARMM36 was used for all protein atoms^72–74^. Small molecule ligand atoms were parameterized with ParamChem/CGenFF atom types^75–77^ and partial atomic charges from the MATCH atom parameterization tool^78^. The TIP3P water model was used to represent water^79^. Initial protein complex coordinates were taken from PDBIDs 1I06^80^, 4CC5^53^, 4R1Y^54^, 2ZFF^55^, and 6HVI^56^ for MUP1, DNA ligase, c-Met, thrombin, and PFKFB3, respectively. Protonation states of titratable residues at a pH of 7.0 were determined with the assistance of MolProbity^81^ and ProPKa^82^. Protein systems were prepared and solvated using the CHARMM-GUI webserver^83^ and cubic water boxes were constructed with a 10 Å buffer between solute atoms and box edges. Enough ions to neutralize the system and create a 0.1 M NaCl solution were added. Small molecule structure files for MUP1, DNA ligase, thrombin, and PFKFB3 ligands were constructed manually using UCSF Chimera^84^. Published structure files were used as initial coordinates for the c-Met compounds^5^. Alchemical functional groups were created as multiple topology models, with explicit atoms for every unique functional group, using the msld-py-prep utility^85^. Cubic unbound ligand solvent boxes were constructed with the convpdb.pl tool from the MMTSB toolset^86^, with a 12 Å buffer between solute atoms and box edges. Starting psf topology and pdb coordinate files were generated with the CHARMM molecular simulation package prior to running LaDyBUGS in OpenMM^65,66^. To track alchemical transformations along connective edges between ligand end states, a series of discrete *λ* states were created for each system following the procedure used for d-GSλD^47^. In OpenMM, the CHARMM-generated psf and pdb files were loaded in with the CharmmPsfFile and CharmmParameterSet classes. A nonbonded lookup table was generated to handle CHARMM’s NBFIX nonbonded parameter exceptions, and custom nonbonded forces were written to facilitate *λ* scaling of all alchemical functional groups. These custom nonbonded forces included CHARMM’s force switching and *λ*D-based soft-core potentials^41,87^. All LaDyBUGS simulations were performed at 25 °C and 1 atm in the isothermal-isobaric ensemble. In OpenMM, this was accomplished with a Monte Carlo barostat^88,89^ and a Langevin integrator^90^ with a friction coefficient of 10 ps^−1^. An integration time step of 2 fs was used, facilitated by constraining all hydrogen to heavy atom bond lengths with the SHAKE algorithm^91^. Periodic boundary conditions were employed, and force switching was used to gradually smooth nonbonded forces to zero between 10 and 12 Å^87^. During a LaDyBUGS simulation, trajectory frames were saved at the end of a Gibbs sampler step, if an alchemical end state was sampled. VMD^92^ and PyMOL^93^ were used to visualize and analyze simulation trajectories.

### LaDyBUGS free energy calculations

Ligands in the five test systems were grouped together as follows: 6 MUP1 ligands were sampled collectively, 7 DNA ligase ligands were sampled collectively, 11 c-Met ligands were grouped into two sets of 6 ligands each, 11 thrombin ligands were grouped into two sets of 6 ligands each, and 10 PFKFB3 ligands were grouped into two sets of 6 and 5 ligands, respectively. For c-Met, thrombin, and PFKFB3 calculations, a common reference compound was featured in each group to connect the two datasets (Fig. 3, red-boxed reference molecules). A symmetric lambda spacing (Δ*λ*) of 0.1 along connective edges was used for all transformations. For LaDyBUGS calculations performing single-site alchemical perturbations only, the number of total *λ* states (*N*_*λ*_) scales quadratically with the number of ligands analyzed (*N*_*s*_), as shown in Eq. (5). As a result, 95 *λ* states were used in LaDyBUGS calculations analyzing 5 ligands, 141 *λ* states were used to evaluate 6 ligands, and 196 *λ* states were used to evaluate 7 ligands collectively. Though this work only investigates single-site perturbations, multisite perturbations are feasible as well, as demonstrated with d-GSλD^47^.

5 $${N}_{{{{{{\rm{\lambda }}}}}}}={N}_{s}+\frac{{N}_{s}({N}_{s}-1)}{2}\left(\frac{1}{\Delta {{{{{\rm{\lambda }}}}}}}-1\right)$$

For each LaDyBUGS calculation, the chemical system was subjected to 1000 steps of energy minimization at a random fixed *λ* state, followed by 5000 steps of MD equilibration to briefly relax the system. The workflow in Fig. 9 was then followed, with iterative sampling of *P*(*X* | *λ*) and *P*(*λ* | *X*) conditional distributions. MD was run for 100 time steps (200 fs) to sample *P*(*X* | *λ*), and biases were updated after every *P*(*λ* | *X*) sample was taken. After 1000 Gibbs sampler steps (200 ps), a FastMBAR calculation was performed, and the $\Delta {G}_{t=u}$ results were used to update the biases according to Eq. (4). Gibbs sampling with bias updates then resumed. LaDyBUGS simulations were run for 15 ns each, during which FastMBAR was called 75 times for bias refinement (every 1000 Gibbs sampler steps). Simulations were run in triplicate for a total of 45 ns of simulation time expended per compound group. FastMBAR was used to collate data from all duplicate runs to yield the final free energy results, and bootstrapping was used to provide an estimate of precision. To investigate the effects of running LaDyBUGS for shorter or longer, simulations were also run for 5 ns and 25 ns each, respectively. Computed relative free energy differences (ΔΔ*G*_comp_) were converted into absolute free energy differences (Δ*G*_comp_) for comparison to experiment (Δ*G*_expt_) with Eq. (6)^16,49^.

6 $$\Delta {G}_{{{{{{\rm{comp}}}}}}}=\Delta \Delta {G}_{{{{{{\rm{comp}}}}}}}-\left(\frac{\sum \Delta \Delta {G}_{{{{{{\rm{comp}}}}}}}}{n}-\frac{\sum \Delta {G}_{{{{{{\rm{expt}}}}}}}}{n}\right)$$

### TI/MBAR free energy calculations

For each chemical system, pairwise perturbations were run between a reference ligand, highlighted with a red box in Fig. 3, and all other ligand analogs for a protein system. This perturbation approach has often been called a “star map” (Fig. 2); redundant calculations for cycle closure were not performed to maximize TI/MBAR efficiency^18–20^. Alchemical transformations were accomplished over 11 *λ* windows with a Δ*λ* schedule of 0.1. For each *λ* window, the chemical system was subjected to 1000 steps of energy minimization and 5000 steps of MD equilibration. MD simulations were then run for 5 ns per *λ* window, and configurations were saved every 100 time steps (200 fs) for a subsequent FastMBAR analysis. Similar to LaDyBUGS, each calculation was run in triplicate for a total of 165 ns of sampling per pairwise perturbation. For systems with larger perturbations, specifically thrombin and PFKFB3 which mutate whole aromatic rings of 6–12 heavy atoms, we observed poor rotational sampling of the perturbed aromatic rings. Therefore, additional sampling of alternative rotational states was performed by manually flipping the aromatic rings by 180° prior to rerunning the TI pairwise calculations. For thrombin, this required twice the total amount of sampling per transformation (330 ns) to perform perturbations from a symmetric reference phenyl ring to two flipped conformations of every other alchemical substituent. For PFKFB3, perturbations were performed between flipped conformations of both the reference and other alchemical substituents, requiring 4 times the total amount of sampling (660 ns) per transformation. For all systems, configurations from all *λ* windows and duplicates were pooled together and supplied to FastMBAR to estimate a final relative free energy difference and bootstrapped errors. We refer to these results as “TI/MBAR 5 ns per window”. Relative binding free energies were again converted into absolute binding affinities for comparison to LaDyBUGS and experiment. To investigate the effects of running TI/MBAR for longer, *λ* window simulations were also extended and sampled for 15 ns each, referred to as “TI/MBAR 15 ns per window”. These longer simulations required 495–1980 ns of total sampling for a single pairwise perturbation.

### *λ*D free energy calculations

To provide an additional computational dataset for comparison to LaDyBUGS, binding free energies were also calculated with *λ*-dynamics. Simulation parameters and conditions used for LaDyBUGS were similarly employed for *λ*D to provide a close one-to-one comparison. Therefore, the multiple topology models and ligand groupings used for LaDyBUGS were also used for *λ*D. Calculations were run with the CHARMM molecular simulation package utilizing the domain decomposition (DOMDEC) module or the BLaDE engine for GPU accelerated sampling^22,45,65,66^. The Adaptive Landscape Flattening algorithm was used to identify appropriate biasing potentials for each system prior to production sampling^41^. Following conventional λD/ALF protocols, one hundred short 100 ps simulations followed by thirteen longer 1 ns and then five duplicate 5 ns simulations were performed for initial bias identification^41,43,44,47,49,50^. For each system, this required a minimal amount of 48 ns for bias identification with ALF. For DNA ligase and thrombin systems, initial production runs failed to yield good sampling and converged free energy results; thus, production trajectories were reanalyzed with ALF to yield new, refined biases, and the discarded production sampling was added to the overall amount of time required to identify biases for *λ*D. Additional production runs were then performed until satisfactory convergence was observed in both *λ* sampling and the final free energy results.

### Symmetric perturbations

Finally, to demonstrate that no artificial bias is introduced by using dynamic bias updates during a LaDyBUGS calculation, symmetric perturbations were performed. Because the expected answer of Δ*G* = 0.00 kcal mol^-1^ is known, these calculations provide a useful control for evaluating the performance of LaDyBUGS without concern for force field inaccuracies. Utilizing a previous example from d-GSλD^47^, methyl perturbations in two systems were explored to convert toluene into toluene and p-xylene into p-xylene in water. In each system, 1–2 methyl groups were perturbed into identical but atomically distinct substituents at one (toluene) or two (p-xylene) sites, respectively. Simulations were run in triplicate for 25 ns to ensure full convergence of the calculation, and the final free energy results were calculated with FastMBAR.

### Reporting summary

Further information on research design is available in the Nature Portfolio Reporting Summary linked to this article.

### Supplementary information

Supplementary Information

Reporting Summary

### Source data

Source Data

## Data Availability

The data that support the findings of this study are available from the corresponding author upon request. Source data are provided with this paper.

## Code availability

Scripts to setup and run LaDyBUGS simulations in OpenMM and pyCHARMM are available at github.com/Vilseck-Lab/LaDyBUGS and Zenodo^94^.
