# Supplementary material for: Fast free energy estimates from λ-dynamics with bias-updated Gibbs sampling
Source: Nat Commun. 2023 Dec 21;14:8515. doi: 10.1038/s41467-023-44208-9 (PMC10740020; doi:10.1038/s41467-023-44208-9)
Supplement: Supplementary file 1 — Supplementary Information [file 41467_2023_44208_MOESM1_ESM.pdf]

Supplementary Information

Fast free energy estimates from λ-dynamics with bias-updated Gibbs sampling

Michael T. Robo,[1,2,6] Ryan L. Hayes,[3,4] Xinqiang Ding,[5,7] Brian Pulawski,[1] and Jonah Z. Vilseck*,[1,2]

[1] Department of Biochemistry and Molecular Biology, Indiana University School of Medicine, Indianapolis, Indiana 46202, United States.

[2] Center for Computational Biology and Bioinformatics, Indiana University School of Medicine, Indianapolis, Indiana 46202, United States.

[3] Chemical and Biomolecular Engineering, University of California, Irvine, California 92617, United States.

[4] Pharmaceutical Sciences, University of California, Irvine, California 92617, United States.

[5] Department of Chemistry, Massachusetts Institute of Technology, Cambridge, Massachusetts 02139, United States.

[6] Current address: Michael T. Robo, Indiana Biosciences Research Institute, 1210 Waterway Blvd Ste. 2000, Indianapolis, IN 46202, United States.

[7] Current address: Xinqiang Ding, Department of Chemistry, Tufts University, Medford, MA 02144, United States.

* Corresponding Author (e-mail: jvilseck@iu.edu)

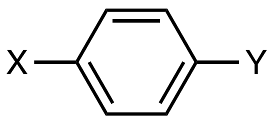

Supplementary Figure 1. Symmetric perturbation test system. The 1,4-benzene chemical system used to test toluene and p-xylene symmetric methyl perturbations (see Table 1 in the main text). Methyl group transformations were explored at site X only for toluene and at both X and Y sites for p-xylene.

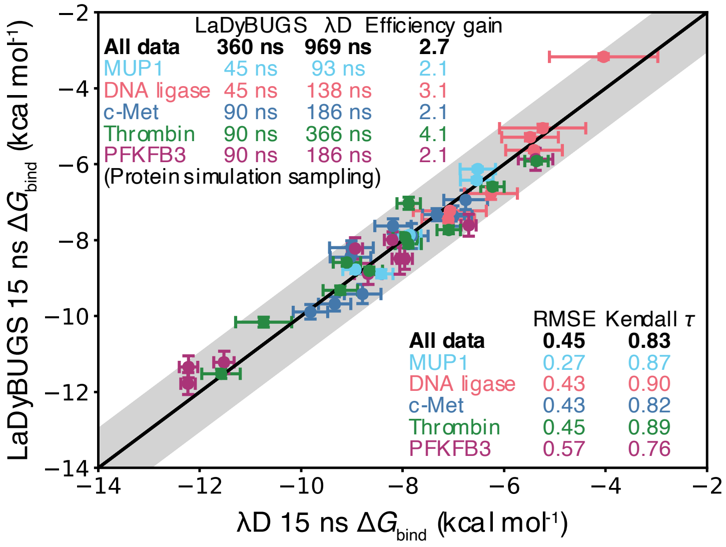

Supplementary Figure 2. Correlation between computed LaDyBUGS 15ns vs. λD 15 ns ΔG_{bind} results. Data points are colored by chemical system (MUP1 in cyan, DNA ligase in red, c-Met in blue, thrombin in green, and PFKFB3 in purple), and bootstrapped uncertainties computed over 3 replicates with FastMBAR are shown as error bars. The center black line represents $y = x$; the shaded gray area represents an error of ± 1 kcal mol^{-1}. Root-mean-square errors, Kendall τ statistics, total amount of sampling, and efficiency gains of LaDyBUGS 15 ns over λD 15 ns in terms of sampling are reported. Source data are provided as a Source Data file.

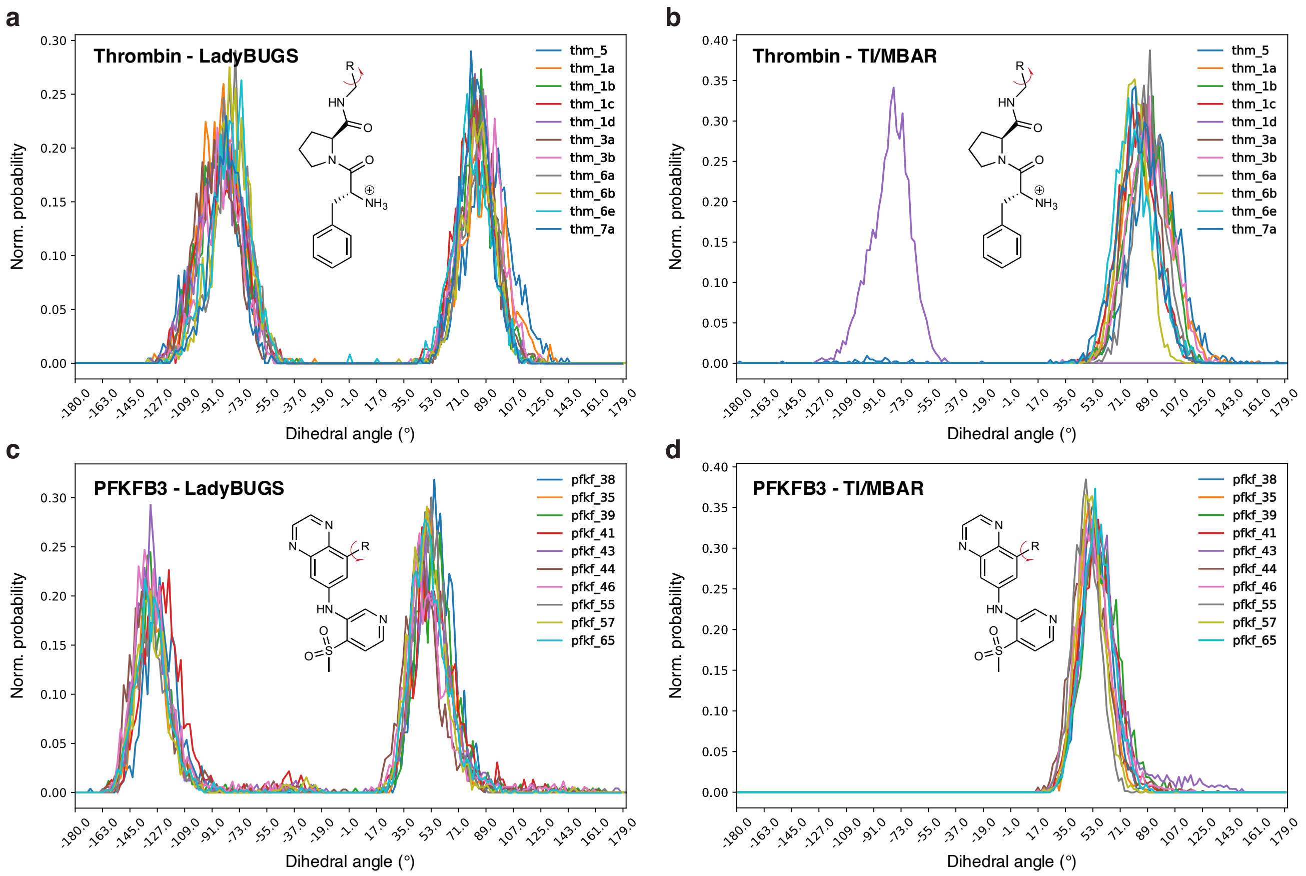

Supplementary Figure 3. Normalized histogram probabilities of sampling R-substituent to ligand core dihedral angles. Dihedral angles are indicated with a red arrow on the inlaid chemical structures. Dihedral sampling for all substituents was analyzed for (**a**) LaDyBUGS 15 ns thrombin, (**b**) TI/MBAR 15 ns per window thrombin, (**c**) LaDyBUGS 15 ns PFKFB3, and (**d**) TI/MBAR 15 ns per window PFKFB3 simulations. TI/MBAR results indicate that only a single conformer was sampled during a complete calculation. In contrast, LaDyBUGS shows equal sampling of both (180° flipped) ring conformations of all aromatic substituents within a single simulation. Source data are provided as a Source Data file.

Supplementary Table 1. Experimental (Expt.) and computed free energies of binding (ΔG_{bind}) with bootstrapped errors[a] (kcal mol^{-1}) for MUP1, DNA ligase, and c-Met benchmark systems with LaDyBUGS, TI/MBAR and λD.[b]

		LaDyBUGS						TI/MBAR[e]				λD[f]	
	Expt.[d]	*5 ns total*		*15 ns total*		*25 ns total*		*5 ns per window*		*15 ns per window*		*15 ns prod.*	
Ligand[c]	ΔG_{bind}	ΔG_{bind}	$\pm\sigma_M$	ΔG_{bind}	$\pm\sigma_M$	ΔG_{bind}	$\pm\sigma_M$	ΔG_{bind}	$\pm\sigma_M$	ΔG_{bind}	$\pm\sigma_M$	ΔG_{bind}	$\pm\sigma_M$
**mup1_7**	-5.64	-6.39	0.11	-6.13	0.06	-6.09	0.05	-6.39	0.07	---	---	-6.52	0.35
**mup1_6**	-6.96	-6.31	0.12	-6.42	0.07	-6.46	0.05	-6.43	0.07	---	---	-6.54	0.32
**mup1_5**	-7.85	-7.83	0.14	-7.86	0.08	-7.86	0.06	-7.68	0.07	---	---	-7.86	0.22
**mup1_4**	-8.20	-8.04	0.15	-8.09	0.08	-8.15	0.06	-7.96	0.11	---	---	-7.89	0.23
**mup1_3**	-9.09	-8.83	0.21	-8.89	0.11	-8.93	0.09	-8.71	0.13	---	---	-8.42	0.23
**mup1_2**	-8.43	-8.77	0.17	-8.78	0.10	-8.68	0.08	-9.00	0.14	---	---	-8.93	0.25
**dnal_4**	-6.50	-7.52	0.13	-7.23	0.07	-7.10	0.06	-7.68	0.06	-7.35	0.05	-7.07	0.72
**dnal_5**	-4.80	-3.38	0.14	-3.17	0.08	-3.03	0.06	-3.45	0.06	-3.60	0.05	-4.04	1.07
**dnal_6**	-5.30	-5.16	0.18	-5.05	0.10	-5.01	0.08	-5.82	0.07	-5.40	0.06	-5.24	0.85
**dnal_7**	-5.25	-5.73	0.13	-5.63	0.07	-5.57	0.06	-5.59	0.06	-5.53	0.05	-5.41	0.56
**dnal_8**	-5.83	-6.43	0.29	-6.77	0.16	-6.87	0.13	-6.30	0.15	-6.60	0.13	-6.26	0.52
**dnal_9**	-6.38	-5.07	0.18	-5.29	0.10	-5.45	0.08	-4.61	0.08	-4.96	0.06	-5.49	0.56
**dnal_4**	-6.54	-7.32	0.14	-7.46	0.08	-7.58	0.06	-7.16	0.06	-7.14	0.05	-7.10	0.52
**cmet_11**	-7.71	-7.35	0.28	-7.33	0.16	-7.32	0.12	-7.10	0.11	-7.18	0.08	-7.33	0.28
**cmet_7**	-7.43	-8.25	0.32	-8.20	0.18	-8.19	0.14	-7.96	0.12	-7.85	0.09	-9.01	0.43
**cmet_10**	-8.69	-9.94	0.34	-9.89	0.19	-9.76	0.14	-10.46	0.13	-10.22	0.09	-9.82	0.34
**cmet_12**	-6.14	-7.51	0.33	-7.32	0.18	-7.12	0.14	-7.30	0.12	-7.27	0.09	-7.04	0.26
**cmet_9**	-8.56	-9.70	0.34	-9.68	0.19	-9.51	0.14	-9.62	0.16	-9.46	0.10	-9.34	0.32
**cmet_8**	-7.17	-7.78	0.34	-7.63	0.19	-7.66	0.14	-8.20	0.15	-7.95	0.10	-8.19	0.36
**cmet_4**	-8.86	-7.51	0.45	-7.37	0.27	-7.37	0.20	-7.72	0.28	-7.64	0.21	-7.00	0.37
**cmet_5**	-9.10	-7.95	0.56	-7.89	0.33	-8.10	0.25	-7.43	0.22	-7.78	0.15	-7.82	0.32
**cmet_13**	-9.10	-9.24	0.43	-9.42	0.25	-9.44	0.18	-9.06	0.14	-9.18	0.10	-8.79	0.36
**cmet_15**	-7.30	-6.76	0.45	-6.94	0.26	-7.09	0.20	-6.54	0.14	-6.77	0.11	-6.76	0.43
**cmet_14**	-10.05	-8.13	0.45	-8.45	0.26	-8.55	0.19	-8.73	0.13	-8.83	0.10	-9.03	0.40

[a] Errors are presented as the standard error of the mean (σ_M).
[b] Source data are provided as a Source Data file.
[c] The chemical structures of each ligand can be found in Fig. 3 of the manuscript.
[d] Supplementary References 1-5
[e] ns sampled per λ window
[f] ns sampled per production (prod.) simulation; does not include time spent identifying biases.

Supplementary Table 2. Experimental (Expt.) and computed free energies of binding (ΔG_{bind}) with bootstrapped errors[a] (kcal mol^{-1}) for thrombin and PFKFB3 benchmark systems with LaDyBUGS, TI/MBAR and λD.[b]

		LaDyBUGS						TI/MBAR[e]				λD[f]	
	**Expt.**[d]	*5 ns total*		*15 ns total*		*25 ns total*		*5 ns per window*		*15 ns per window*		*15 ns prod.*	
**Ligand**[c]	ΔG_{bind}	ΔG_{bind}	$\pm \sigma_M$	ΔG_{bind}	$\pm \sigma_M$	ΔG_{bind}	$\pm \sigma_M$	ΔG_{bind}	$\pm \sigma_M$	ΔG_{bind}	$\pm \sigma_M$	ΔG_{bind}	$\pm \sigma_M$
**thm_5**	-7.58	-5.70	0.13	-5.92	0.08	-5.97	0.06	-5.59	0.07	-5.47	0.04	-5.36	0.23
**thm_1a**	-7.48	-6.28	0.16	-6.59	0.09	-6.71	0.07	-6.28	0.08	-6.08	0.04	-6.23	0.23
**thm_1b**	-8.46	-8.54	0.16	-8.81	0.10	-8.93	0.07	-8.75	0.08	-8.68	0.05	-8.66	0.26
**thm_1c**	-8.56	-8.39	0.17	-8.59	0.10	-8.75	0.07	-9.70	0.09	-9.79	0.05	-9.10	0.27
**thm_1d**	-8.25	-7.89	0.18	-8.10	0.11	-8.22	0.08	-8.56	0.15	-8.40	0.09	-7.89	0.26
**thm_3a**	-8.32	-7.43	0.17	-7.73	0.10	-7.85	0.07	-7.04	0.09	-7.08	0.05	-7.09	0.23
**thm_3b**	-7.86	-7.48	0.27	-7.03	0.16	-7.05	0.13	-6.97	0.15	-6.98	0.08	-7.88	0.23
**thm_6a**	-9.18	-11.64	0.20	-11.52	0.12	-11.31	0.09	-11.67	0.10	-11.71	0.06	-11.58	0.38
**thm_6b**	-8.89	-10.66	0.21	-10.16	0.13	-9.92	0.10	-10.20	0.11	-10.25	0.06	-10.74	0.55
**thm_6e**	-8.91	-9.35	0.19	-9.32	0.11	-9.20	0.08	-9.21	0.09	-9.31	0.05	-9.23	0.34
**thm_7a**	-8.22	-8.35	0.22	-7.93	0.13	-7.80	0.10	-7.73	0.11	-7.95	0.06	-7.95	0.24
**pfkf_38**	-8.76	-7.96	0.37	-7.99	0.21	-7.95	0.16	-7.55	0.42	-7.57	0.23	-8.20	0.16
**pfkf_35**	-10.14	-11.34	0.51	-11.22	0.29	-11.09	0.22	-11.98	0.51	-11.64	0.29	-11.52	0.20
**pfkf_39**	-7.20	-5.78	0.50	-5.87	0.29	-5.86	0.22	-4.95	0.90	-5.42	0.47	-5.38	0.34
**pfkf_41**	-7.39	-8.49	0.76	-8.49	0.41	-8.61	0.33	-8.89	0.66	-8.78	0.37	-8.06	0.15
**pfkf_43**	-8.00	-8.84	0.50	-8.89	0.28	-8.86	0.22	-8.50	0.53	-8.52	0.29	-8.68	0.27
**pfkf_55**	-10.64	-11.91	0.50	-11.78	0.29	-11.66	0.22	-12.25	0.54	-12.02	0.29	-12.23	0.14
**pfkf_44**	-9.95	-8.35	0.48	-8.49	0.28	-8.62	0.21	-8.43	0.65	-8.57	0.34	-7.96	0.15
**pfkf_46**	-7.86	-7.62	0.49	-7.61	0.29	-7.79	0.22	-7.48	0.51	-7.63	0.28	-6.70	0.15
**pfkf_57**	-10.47	-11.47	0.50	-11.34	0.29	-11.09	0.23	-12.11	0.56	-11.64	0.31	-12.22	0.18
**pfkf_65**	-9.49	-8.11	0.49	-8.22	0.28	-8.36	0.22	-7.74	0.57	-8.11	0.31	-8.94	0.15

[a] Errors are presented as the standard error of the mean (σ_M).
[b] Source data are provided as a Source Data file.
[c] The chemical structures of each ligand can be found in Fig. 3 of the manuscript.
[d] Supplementary References 1-5
[e] ns sampled per λ window
[f] ns sampled per production (prod.) simulation; does not include time spent identifying biases.

Supplementary Table 3. Normalized probabilities of transition distances between sampled λ states from all (protein bound) LaDyBUGS benchmark simulations.[a]

Transition Distance	c-Met Group 1	c-Met Group 2	DNA ligase	MUP1	thrombin Group 1	thrombin Group 2	PFKFB3 Group 1	PFKFB3 Group 2
0	0.0548	0.0798	0.0263	0.0321	0.0386	0.0586	0.0757	0.0771
1	0.3700	0.5133	0.1915	0.2408	0.3160	0.4301	0.4750	0.4770
2	0.2372	0.2356	0.1502	0.1774	0.2196	0.2323	0.2227	0.2268
3	0.1349	0.0978	0.1176	0.1287	0.1426	0.1215	0.1062	0.1073
4	0.0761	0.0401	0.0936	0.0940	0.0904	0.0626	0.0519	0.0505
5	0.0435	0.0166	0.0748	0.0704	0.0569	0.0345	0.0262	0.0245
6	0.0266	0.0075	0.0636	0.0551	0.0373	0.0203	0.0147	0.0134
7	0.0179	0.0041	0.0530	0.0440	0.0259	0.0132	0.0090	0.0085
8	0.0125	0.0025	0.0439	0.0351	0.0190	0.0088	0.0058	0.0051
9	0.0086	0.0011	0.0359	0.0278	0.0143	0.0055	0.0038	0.0034
10	0.0060	0.0007	0.0293	0.0216	0.0105	0.0038	0.0025	0.0020
11	0.0039	0.0004	0.0233	0.0162	0.0074	0.0024	0.0016	0.0013
12	0.0028	0.0002	0.0244	0.0159	0.0070	0.0022	0.0017	0.0012
13	0.0022	0.0002	0.0242	0.0151	0.0054	0.0017	0.0013	0.0009
14	0.0017	0.0001	0.0261	0.0144	0.0048	0.0016	0.0014	0.0007
15	0.0008	0.0001	0.0146	0.0083	0.0028	0.0008	0.0005	0.0004
16	0.0003	0.0000	0.0040	0.0016	0.0005	0.0001	0.0000	0.0000
17	0.0002	0.0000	0.0021	0.0008	0.0004	0.0001	0.0000	0.0000
0	0.0001	0.0000	0.0010	0.0005	0.0002	0.0000	0.0000	0.0000
1	0.0001	0.0000	0.0004	0.0002	0.0001	0.0000	0.0000	0.0000
2	0.0000	0.0000	0.0001	0.0001	0.0000	0.0000	0.0000	0.0000

[a] Transitions occurring between FastMBAR calculations were not included. Using a discrete λ schedule of Δλ = 0.1, a maximum travel distance of 20 states is possible. c-Met Group 1 and Group 2 results are plotted on the right-hand side of Fig. 8 in the main manuscript. Source data are provided as a Source Data file.

Supplementary Note 1: Mathematical Proof

A mathematical proof is provided to show that the value of the scalar bias used with LaDyBUGS Gibbs sampling does not affect the FastMBAR calculation. This assumes that the MD simulations run during each Gibbs sampler step reach equilibrium. Thus, unbiased equilibrium energies computed during a LaDyBUGS simulation can be used with FastMBAR for free energy estimation.

To begin, we could assume that a unique thermodynamic state of the system is formed every time a different λ state or a different bias is sampled. For example, in the case where the same λ state is sampled in two consecutive Gibbs sampler steps but with different external biases, those states could be considered independent from one another. This means that for a thermodynamic state *i* with a given λ^i state and external bias E^i, we can determine the overall energy of a configuration of the system ($U^i(X,\lambda^i)$) as:

$$U^i(X,\lambda^i) = V(X,\lambda^i) + E^i \tag{1}$$

where E^i is the external bias applied at state *i* and $V(X,\lambda^i)$ is the energy of the system at a specific λ state with no external bias applied (E = 0):

$$V(X=(x_0,\{x\}),\lambda^i) = V_{\text{env}}(x_0) + V_{\text{ss}}(X,\lambda^i) + V_{\text{MS}}(X,\lambda^i) \tag{2}$$

V_{SS} and V_{MS} are obtained from equations 2 and 3 in the main manuscript. By using equation 1 to determine the energy of each state at each configuration, and assuming each configuration is an equilibrium sample from its corresponding state, a table of states and configurational energies can be created as input for an FastMBAR free energy analysis. Because configurations are generated with a unique bias that changes every Gibbs sampler step with LaDyBUGS, the resulting FastMBAR input would scale according to $O(\text{n}^2)$ with the number of samples taken.[6] To solve for the relative free energy of each state *i*, we can use the FastMBAR equation (equation 3), which has been previously shown to be an alternative method of solving the MBAR equation.[7]

$$\sum_{j=1}^{M}\sum_{k=1}^{L_j}\frac{\exp\left[-\left(U^i\left(X_j^k,\lambda^i\right)+b_i\right)\right]}{\sum_{l=1}^{M}\exp\left[-\left(U^l\left(X_j^k,\lambda^l\right)+b_l\right)\right]} = L_i \tag{3}$$

M is the number of states, L_i is the number of times state *i* is sampled, and b_i is the internal bias of state *i* that is used to ensure the relative populations of *M* states match the free energies of the generalized ensemble. In the FastMBAR approach, a convex function is fashioned from equation 3, which is then minimized to solve for the values of b_i for each state. These values are then used to calculate the relative free energies of the system in the *M* states (G_i^*) using equation 4,

$$G_i = G_i^* + b_i = -\ln\left(\frac{L_i}{\sum_{l=1}^{M} L_l}\right) \tag{4}$$

where G_i represents the relative free energy of the ith state in the generalized ensemble.
Because each state is sampled once, and only once, we can simplify equation 3 into equation 5, where L_i = 1 in all cases.

$$\sum_{j=1}^{M}\sum_{k=1}^{L_j}\frac{\exp\left[-\left(U^i\left(X_j^k,\lambda^i\right)+b_i\right)\right]}{\sum_{l=1}^{M}\exp\left[-\left(U^l\left(X_j^k,\lambda^l\right)+b_l\right)\right]}=1 \qquad (5)$$

Now let us consider two states α and β that share the same λ state ($\lambda^\alpha = \lambda^\beta = \lambda^0$) but were sampled with different external biases, E^α and E^β. Because equation 5 reaches the same value (unity) for all states, we can assert the equivalence shown in equation 6.

$$\sum_{j=1}^{M}\sum_{k=1}^{L_j}\frac{\exp\left[-\left(U^\alpha\left(X_j^k,\lambda^\alpha\right)+b_\alpha\right)\right]}{\sum_{l=1}^{M}\exp\left[-\left(U^l\left(X_j^k,\lambda^l\right)+b_l\right)\right]}=\sum_{j=1}^{M}\sum_{k=1}^{L_j}\frac{\exp\left[-\left(U^\beta\left(X_j^k,\lambda^\beta\right)+b_\beta\right)\right]}{\sum_{l=1}^{M}\exp\left[-\left(U^l\left(X_j^k,\lambda^l\right)+b_l\right)\right]} \qquad (6)$$

Using equation 1, we can substitute into equation 6 to generate equation 7:

$$\sum_{j=1}^{M}\sum_{k=1}^{L_j}\frac{\exp\left[-\left(\mathrm{V}\left(X_j^k,\lambda^0\right)+b_\alpha+E^\alpha\right)\right]}{\sum_{l=1}^{M}\exp\left[-\left(U^l\left(X_j^k,\lambda^l\right)+b_l\right)\right]}=\sum_{j=1}^{M}\sum_{k=1}^{L_j}\frac{\exp\left[-\left(\mathrm{V}\left(X_j^k,\lambda^0\right)+b_\beta+E^\beta\right)\right]}{\sum_{l=1}^{M}\exp\left[-\left(U^l\left(X_j^k,\lambda^l\right)+b_l\right)\right]} \qquad (7)$$

If $\lambda^\alpha = \lambda^\beta = \lambda^0$ and equation 7 is true, then equation 8 must also be true:

$$b_\alpha + E^\alpha = b_\beta + E^\beta = b(\lambda^0) \qquad (8)$$

where $b(\lambda^0)$ is the bias of lambda state λ^0 as if no external bias were applied at all ($E = 0$). The implication of equations 7 and 8 is that different external biases E^i will be automatically counterbalanced by the FastMBAR internal biases b_i. Since we are only interested in the relative free energies of the individual λ^i states at equilibrium (E = 0), we can arbitrarily set all external biases to zero (E^i = 0.0), pool all i^{th} states that share the same λ state together, and treat them as multiple counts of the same λ state as input for FastMBAR, exactly like we would for d-GSλD or TI/MBAR sampling. Thus, under the previously stated assumptions, the set of configurations obtained from LaDyBUGS sampling with a dynamic external bias can be treated in the same manner as a set of configurations obtained using a static external bias with d-GSλD.

From a practical perspective, this creates two advantages. First, the function used to calculate an external bias for LaDyBUGS sampling does not impact the FastMBAR calculation for determining relative free energy differences between all λ states. As equations 7 and 8 show, the effects of an external bias will be automatically counteracted by the internal bias calculated within FastMBAR, so any arbitrary external bias may be freely used. Second, for Gibbs sampling of discrete λ states, bias optimization is unnecessary. A self-correcting dynamic external bias can be used to ensure smooth sampling over all states. No prior simulation time is needed to pre-calculate free energy differences between λ states to create a static bias prior to production sampling.

Supplementary References

1. Howard, S. *et al.* Fragment-Based Discovery of 6-Azaindazoles as Inhibitors of Bacterial DNA ligase. *ACS Med. Chem. Lett.* **4**, 1208–1212 (2013).
2. Steinbrecher, T. B. *et al.* Accurate Binding Free Energy Predictions in Fragment Optimization. *J. Chem. Inf. Model.* **55**, 2411–2420 (2015).
3. Dorsch, D. *et al.* Identification and optimization of pyridazinones as potent and selective c-Met kinase inhibitors. *Bioorg. Med. Chem. Lett.* **25**, 1597–1602 (2015).
4. Baum, B. *et al.* More than a simple lipophilic contact: a detailed thermodynamic analysis of nonbasic residues in the s1 pocket of thrombin*. J. Mol. Biol.* **390**, 56-69 (2009).
5. Boutard, N. *et al.* Discovery and Structure-Activity Relationships of N-Aryl 6-Aminoquinoxalines as Potent PFKFB3 Kinase Inhibitors. *ChemMedChem* **14**, 169-181 (2019).
6. Shirts, M. R. & Chodera, J. D. Statistically optimal analysis of samples from multiple equilibrium states. *J. Chem. Phys.* **129**, 124105–124105 (2008).
7. Ding, X., Vilseck, J. Z. & Brooks, C. L. III A Fast Solver for Large Scale Multistate Bennett Acceptance Ratio Equations. *J. Chem. Theory Comput.* **15**, 799–802 (2019).
